# Early fitting in cochlear implant surgery: preliminary results

**DOI:** 10.1007/s00405-023-08076-9

**Published:** 2023-07-07

**Authors:** Arianna Soncini, Sebastiano Franzini, Francesca Di Marco, Pasquale Riccardi, Andrea Bacciu, Enrico Pasanisi, Filippo Di Lella

**Affiliations:** 1 Department of Medicine and Surgery, University of Parma, via Gramsci 14, 43121 Parma, Italy; 2 Advanced Bionics Italia, Via Privata Raimondo Montecuccoli, 30, 20147 Milan, MI Italy

**Keywords:** Cochlear implant, Activation, Electrophysiology, Impedance, Most comfortable loudness, Early fitting

## Abstract

**Purpose:**

Cochlear implants are usually activated 3–5 weeks after surgery; to date, no universal protocol exists regarding switch on and fitting of these devices. The aim of the study was to assess safety and functional results of activation and fitting of cochlear implant within 24 h following surgery.

**Methods:**

In this retrospective case–control study, 15 adult patients who underwent cochlear implant surgery, for a total of 20 cochlear implant procedures were analyzed. In particular, clinical safety and feasibility were investigated by examinating patients at activation and at each follow-up. Values of electrodes’ impedance and most comfortable loudness (MCL) were analyzed from the time of surgery to 12 months after activation. Free-field pure tone average (PTA) was also recorded.

**Results:**

No major or minor complications were reported and all patients could perform the early fitting. Activation modality influenced impedance values only in the short term but the differences were not statistically significant (*p* > 0.05). Mean MCL values in the early fitting group were lower than MCL of the late fitting in all follow-up sessions, and the difference was statistically significant (*p* < 0.05). The mean PTA was lower in the early fitting group but the difference was not statistically significant (*p* < 0.05).

**Conclusions:**

Early fitting of cochlear implants is safe, allows for an early rehabilitation and can have possible beneficial effects on stimulation levels and dynamic range.

## Introduction

Technological developments over the last decades have brought significant changes in both the function and physical dimensions of cochlear implants (CI) with impact on the surgical approach required for device placement. The currently used minimal incisions and soft tissue dissection reduce the aesthetic impact of the surgery and minimize wound complications [1–3]. The risks of CI surgery are nowadays reduced to a minimum and most patients are discharged the day after surgery. Activation of the device is usually scheduled 3–5 weeks from discharge to ensure optimal wound healing and oedema reduction; nonetheless, no universal protocol exists regarding timing of activation.

The aim of the study was to assess safety and functional results of early activation and fitting of cochlear implant within 24 h following surgery.

## Patients and methods

In this case–control study, a retrospective analysis was performed to identify the patients who underwent CI surgery between January 2016 and December 2021. Inclusion criteria were: (1) adult patients, (2) primary surgery, and (3) normal inner ear anatomy and postoperative follow-up of at least 1 year. The otological surgical database of the Authors’ tertiary referral University Hospital was retrospectively reviewed. Medical charts, imaging and surgical reports of all patients who underwent cochlear implant surgery were analyzed. Details regarding patient demographics, medical and surgical charts as well as electrophysiological and functional results at last available follow-up were collected in a retrospective manner. All clinical investigations were conducted according to the principles expressed in the Declaration of Helsinki; study protocol was submitted and authorized by the local Ethical Committee. All subjects gave their written consented to participate.

### Fitting procedure

For all the subjects the Optima S stimulation strategy was adopted [4] and devices were fitted by the same clinical specialist (FDM) in every follow-up visit. Patients activated within 24 h from surgery were advised to use the device only a few hours a day (i.e., 2 h in the morning and 2 h in the afternoon) for the first month. During the first month after initial fitting, patients in both EF and LF groups are instructed to increase their global MCL by 10 CU steps when a decrease in loudness is perceived.

### Clinical end-points

Different clinical end-points were considered to investigate the safety and feasibility of the early activation. All the patients were examined by a physician the day after surgery, at activation and at each follow-up to evaluate the wound status (oedema, dehiscence, infection), the receiver location, the presence of pain/discomfort and other possible audiovestibular symptoms (i.e., vertigo or tinnitus).

### Audiological and electrophysiological parameters

Impedance history of each electrode of the CI users between 2016 and 2021 was retrospectively analyzed. The early fitting included the following follow-up measures: intraoperative, activation (within 24 h after surgery), 1, 3, 6, 9 and 12 months from activation. In the late fitting group, patients were evaluated intraoperatively, at activation and at 1, 3, 6, 9 and 12 months after activation.

The same follow-up was applied for the analysis of the most comfortable loudness (MCL) evolution of all electrodes.

Free field pure tone average (PTA), defined as average of hearing threshold levels at the frequencies 500, 1000, 2000 and 4000 Hz, was evaluated at each follow-up.

### Data analysis

Statistical analysis was performed using Microsoft Excel 365 software for Microsoft Windows. Descriptive statistics for both continuous variables (means and standard deviations) were assessed. Variables were compared by means of parametric tests, in particular, the paired-sample *t* test which was performed on a per electrode basis. *p* values < 0.05 were considered significant for all tests.

## Results

The population of the study included 15 patients (7 ♂, 8 ♀), for a total of 20 CIs. The study group included 10 CIs with activation and initial fitting performed within 24 h after surgery (early fitting—EF). In the control group, which included 10 CIs, the sound processor was fitted at 3–6 weeks after surgery (late fitting—LF). Participants received their CI at the Authors’ institution Ospedale Maggiore di Parma, Italia. For 14 patients, surgery was carried out using a standard limited mastoidectomy with posterior tympanotomy approach, while 1 patient of the study group underwent subtotal petrosectomy for simultaneous removal of epitympanic cholesteatoma. In all cases, a slow controlled (i.e., 2 min) insertion of the array was performed after incision of the round window membrane.

In two patients of the EF cohort, bilateral cochlear implantation was carried out, as well as in the LF cohort. Moreover, one patient with bilateral CIs underwent LF for the first device and EF for the second device. Average age of the patients at the time of surgery was 44.6 ± 15.3 years old (LF = 48.6 years old, EF = 40.6 years old). The mean follow-up of the patients was 36.4 months (14.7–71.7 months); mean follow-up of was 26.3 months and 46.4 months for the EF and LF groups, respectively. The demographic characteristics of the two groups are shown in Table 1. All the CIs were produced by Advanced Bionics Co. and the following models were implanted: HiRes ULTRA MS, HiRes ULTRA3D MS, HiRes ULTRA3D SJ, HiRes 90K Advantage MS.

**Table 1:** Demographic characteristics and mean pure tone average (PTA) of the patients

	Early fitting	Late fitting
Ears, *n*	10	10
Age		
Mean, years	40.6 ± 14.5	48.6 ± 14.8
Side left:right	4:6	3:7
Follow-up		
Mean, months	26.3	46.4
Range, months	14.7–45.0	20.8–71.7
PTA at last follow-up, dB	44.1	45.0

### Safety and feasibility

No major intraoperative or postoperative complications were reported, in particular, none of the patients reported pain and the early activation was possible for all selected patients. The first fitting session took place within 24 h after surgery for the EF group and at a mean of 30 days (range 22–36 days) after surgery for the LF group. At the 12-month follow-up, no displacement of the receiver was observed and the healing of the wound was regular in both groups. Moreover, no patient reported pain or other symptoms.

### Electrode impedance

**Fig. 1:**
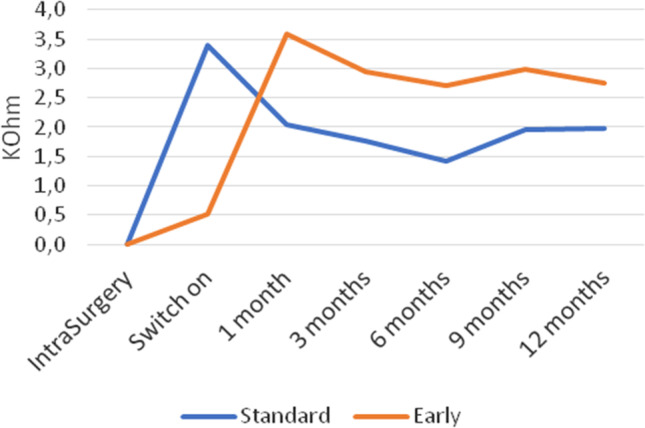
Mean cumulative relative impedance growth (electrodes 1–16) expressed in kOhm at each follow-up for study and control groups, by setting the activation value equal to 0. Measures were performed intraoperatively, at activation and at 1–6–12 months after surgery

**Fig. 2:**
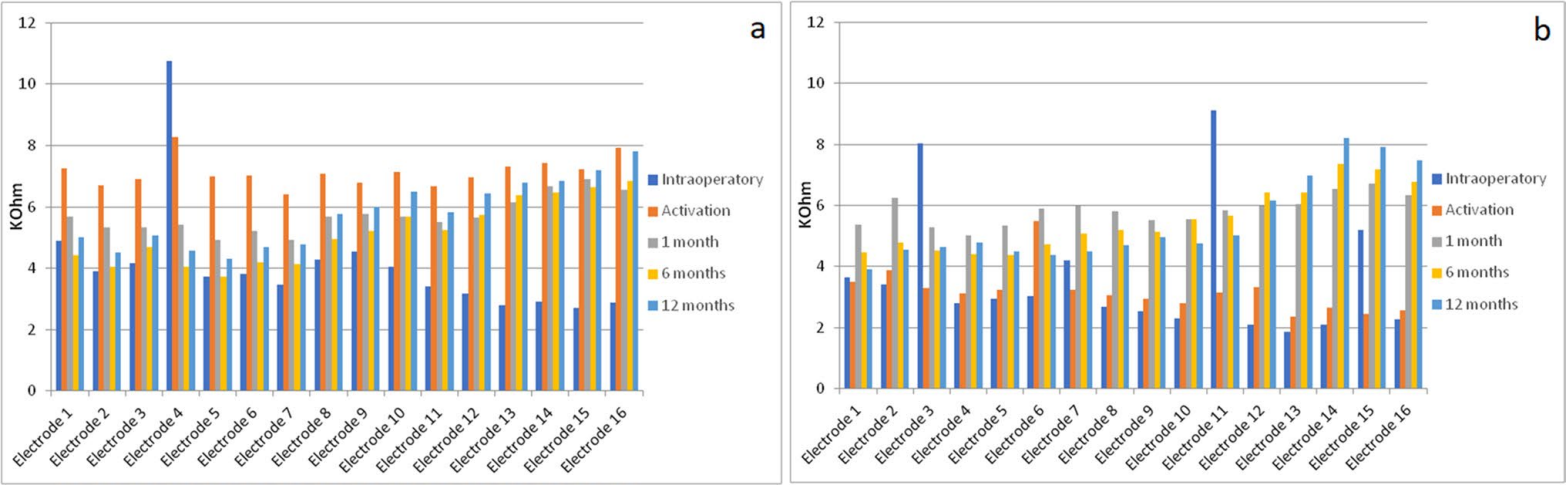
Single electrode mean impedance values at each follow-up in late fitting [LF, (**a**)] and early fitting [EF, (**b**)] groups

In the short term, the impedance trend was influenced by the activation mode; in the late fitting group, the value rose until activation and then lowered 1 month after implant use. In contrast, in the early activation group, the impedance increased from activation to 1 month. From the first month onwards, numbers showed a similar trend for the two groups with values that do not deviate from each other. Differences were not statistically significant (*p* > 0.05), and therefore, impedances were not influenced by activation modality (Figs. 1, 2).

### Most comfortable loudness

Mean MCL values in the early fitting group were lower than MCL of the late fitting in all the follow-up sessions, and the difference was statistically significant (*p* < 0.05).

### PTA

The global mean PTA was 44.5 ± 6.5 dB. As shown in Table 1, in the EF group, the mean PTA was 44.1 dB (37.5–48.8), while in the LF group, it was 45.0 dB (38.8–61); the difference was not statistically significant (*p* < 0.05).

## Discussion

This study retrospectively evaluated different elements to investigate safety, feasibility and the effects on electrophysiological parameters of activation of cochlear implants within 24 h from surgery. The results of the patients who underwent early activation were compared to the results of a control group, composed of patients who underwent activation from 3 to 5 weeks after implantation.

Definition of early activation varies in the literature. Some authors define it as the activation of the cochlear implant within 1 day after surgery [5–9], other as the activation from 1 to 8 days after surgery [10], or from 2 to 7 days after implantation [11]. Activation at 5 days after surgery [12] or activation within 14 days after CI [13] has also been reported.

In this study, there were no major intraoperative or postoperative complications, such as oedema, dehiscence, infection, or pain; no migration of the receiver nor other symptoms such as vertigo or tinnitus were observed or reported. These results are consistent with other studies which confirm early activation to be safe and feasible in the absence of major complications or adverse events in the majority of the patients [5, 6, 10–13]. In our experience, all the 8 patients (10 CIs) selected for early fitting well tolerated the procedure and no side effects were reported. In other studies, some patients had to postpone activation either, because initial wound swelling did not allow the coupling between the processor and the receiver [13], or because of the occurrence of pain [11]. Magnet strength did not need adjustments in early activation group and this result is consistent with other experiences [5, 13], although it is also reported that in some cases, the magnet strength needs to be reduced over time in patients who undergo early activation [11].

Early activation can thus be considered safe and feasible and it does not increase the risk of major complications. Nonetheless, it is important to monitor the healing of the wound of each patient and to consider the possibility of postponing the activation in case of oedema or pain reported from the patient. Another important aspect is the selection of the magnet, which should be strong enough to allow the coupling of the processor with the receiver, without causing skin necrosis or pain.

Impedance is defined as resistance to the electric current and is measured in kOhm. It is given by the sum of the fixed impedance (intrinsic impedance of the materials constituting the device electrical cable and electrode) and a variable impedance (polarization impedance, due to the effect of the perilymph between the electrodes and the cochlear wall and the fibrosis developing around the electrode surface) [14].

Electrode impedance increases after implantation of the array due to intracochlear formation of new bone and tissue around the electrode [14–16]. Moreover, it seems that fibrosis leads to a change in composition of perilymph or extracellular fluid adjacent to the electrodes with consequent increase in impedance values [17]. Some studies found that impedance values decreased after initial activation and different hypotheses were considered, among which the reorganization of the newly formed tissue sheath due to electrical current or the resumed sensitivity of neuronal membranes to the electrical stimulation [18, 19].

According to these mechanisms, impedances in the early activation group were expected to be lower, but the difference between the two activation modalities was not significant in Authors’ series.

As shown in Fig. 1, impedance values trend was different when comparing the two activation modalities only in the first weeks after surgery. In the EF group, impedances remained stable between surgery and activation, increased from activation to 1 month and then reached a stable level. Impedances in the LF group showed an initial steep increase from implantation to activation and then reached a stable level from 1 month after activation. Statistical analysis revealed that the difference of the impedances between the two groups was not significant even in the long term.

Similar results were reported in a previous study [10], in which electrode impedances increased in the first month after activation in the early fitting group, and at the 1-month follow-up after activation, there was no difference in impedances between the EF and LF groups. Alsabellha et al. [12], in accordance with our findings, reported that although impedances of the EF group were lower than those of the LF group, they were similar at 1-month follow-up.

The same trend of impedances in early activation was shown in another study [5], where it was observed that impedance values in patients who underwent early activation were higher at 1 month compared to activation; the same author concluded that the variable impedance component seems not to be influenced by the activation method.

**Fig. 3:**
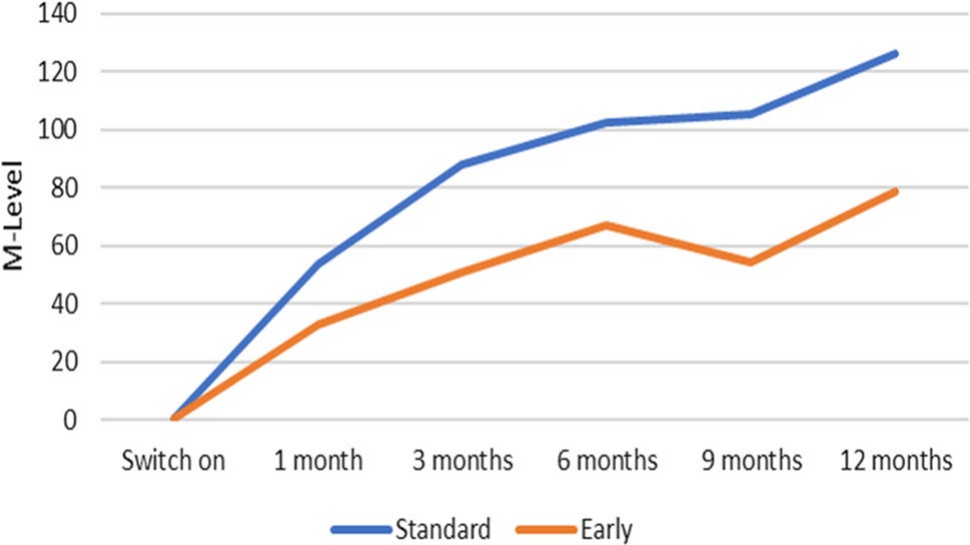
Mean cumulative relative MCL growth (electrodes 1–16) expressed in current unit at each follow-up for study and control groups, by setting the activation value equal to 0

**Fig. 4:**
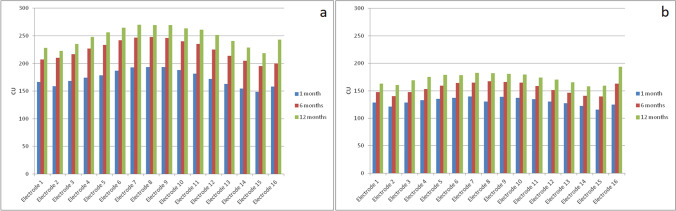
Average MCL per electrode in [LF, (**a**)] and early fitting [EF, (**b**)] groups at 1–6–12 months after surgery

MCL is measured in current units (CU) and it represents the intensity at which the patient obtains a comfortable hearing [20]; it is a behavioral measure and it is estimated using verbal feedbacks from the patient in response to the modifications applied to the intensity of the stimulus. The analysis of the MCL data surprisingly showed a statistically significant difference depending on activation modality; although MCL levels of both groups increased at the follow-up sessions compared to the initial activation, the values of the EF group were repeatedly and consistently lower than those of the LF group. In the long term (i.e., 12-month follow-up), a mean global difference of 40 CUs was observed (Fig. 3) (*p* < 0.05) with relative growth of 120 CUs in the LF group and 80 CUs in the EF group. Per-electrode analysis showed lower CU values in all electrodes at all follow-up sessions in the EF versus LF group (Fig. 4).

Explanations for these findings could be that MCL measures a different aspect of the CI/neural interface with respect to that measured by impedance; the early transmission of current can reduce the damages induced by surgical trauma and prevent further deterioration of residual nerve fibers [21].

Only few studies examined the influence of the activation modality on MCL. Among these, Alsabellha et al. [12], noted that MCL levels increase at the follow-up sessions and they are lower in the early activation group, but differently from our study the difference from the standard activation group is not significant. Another study [7] reported that MCL increased at the follow-up sessions compared to the switch-on session, but in contrast with our results the MCL was higher in the early fitting group. The evolution of MCL at 1 month from activation was also analyzed in another study [5], where it was observed a progressive increase in MCL values compared to activation in a group of patients who underwent early fitting. In the same study, it was also analyzed the influence of impedance levels on stimulation levels and it appeared that the former were not predictable of the stimulation levels.

The reduction of MCL levels may have many advantages, especially in the long term. First of all, a lower MCL guarantees a wider electrical dynamic range, with reduced risks of side effects (i.e., facial nerve stimulation) and possibly better hearing results [22]. Considering that many patients who undergo cochlear implantation are young, a lower initial MCL assures a wider margin of maneuver on current levels during the lifespan. From a more practical point of view, another advantage of a lower MCL and thus a lower intensity of current, could be enhanced battery life.

Main weakness of this study is its retrospective nature and limited number of patients; on the other hand, statistical significance derived from a high number of observations on each electrode with consistent and repeated findings in the long term. Moreover activation, fitting and electrophysiological evaluation was performed by the same professional removing possible interoperator biases.

## Conclusions

The early fitting of cochlear implants within 24 h from surgery is a safe and feasible procedure that can be performed in the majority of the patients. Main advantages include reduced interval between surgery and rehabilitation and possible long term beneficial effects on stimulation level and dynamic range.

## Data Availability

Anonymized raw data are available on request to the corresponding author.
